# Hox Transcription Factors: Modulators of Cell-Cell and Cell-Extracellular Matrix Adhesion

**DOI:** 10.1155/2014/591374

**Published:** 2014-07-21

**Authors:** Yasushi Taniguchi

**Affiliations:** Division of Basic Molecular Science and Molecular Medicine, School of Medicine, Tokai University, Isehara, Kanagawa 259-1193, Japan

## Abstract

*Hox* genes encode homeodomain-containing transcription factors that determine cell and tissue identities in the embryo during development. *Hox* genes are also expressed in various adult tissues and cancer cells. In *Drosophila*, expression of cell adhesion molecules, cadherins and integrins, is regulated by Hox proteins operating in hierarchical molecular pathways and plays a crucial role in segment-specific organogenesis. A number of studies using mammalian cultured cells have revealed that cell adhesion molecules responsible for cell-cell and cell-extracellular matrix interactions are downstream targets of Hox proteins. However, whether Hox transcription factors regulate expression of cell adhesion molecules during vertebrate development is still not fully understood. In this review, the potential roles Hox proteins play in cell adhesion and migration during vertebrate body patterning are discussed.

## 1. Introduction

Homeobox genes (*Hox* genes) were initially identified in* Drosophila* through genetic mutations that resulted in transformations of one body segment to another—so-called homeotic transformations [1]. Homeoboxes are 183-bp sequences that encode highly conserved 61-amino-acid homeodomains with helix-turn-helix motifs that are responsible for binding specific DNA sites [2]. Homeodomain proteins are transcription factors that modulate expression levels of their target genes [3, 4]. In amniotes including mammals and birds, 39* Hox* genes are arranged in four clusters on different chromosomes. Numerous genetic analyses of loss- and gain-of-function mutations in mice have revealed that* Hox *genes play pivotal roles in determining the identities of cells and tissues in the developing embryo. In adult animals,* Hox* expression is required for the proliferation and differentiation of hematopoietic cells [5–7] and renewal of the endometrium [8–10]. Because* HOX* genes are frequently deregulated in human cancer cells, HOX proteins can be used as both diagnostic markers and therapeutic targets for malignant tumors [11].

Recent studies have converged on identifying downstream Hox target genes. Genome-wide techniques such as microarrays and chromatin immunoprecipitation have been used to identify Hox-regulated genes in* Drosophila*, mice, and cultured cells. The Hox target genes identified thus far are very diverse with regard to their roles in cellular identity and function. The proteins encoded by the target genes are involved in transcriptional regulation, signal transduction, cell shape, and cell adhesion and migration, as well as in the cell cycle and cell death [12–15]. However, the diverse mechanisms of Hox regulation pose a challenge for elucidating the exact mechanisms by which Hox proteins determine cell identities and where in the molecular cascade they exert their effects. The mechanisms by which Hox transcription factors regulate cellular events are not fully understood.

Since the mouse neural cell adhesion molecule (N-CAM), a mediator of cell adhesion in nervous system tissues during embryonic development, was first identified as a Hox target [16], a number of studies have reported that other cell adhesion molecules, such as cadherins and integrins, are downstream targets of Hox proteins. Cadherins constitute a large superfamily of transmembrane glycoproteins that mediate calcium-dependent intercellular adhesion in most tissues and play important roles in a wide variety of cellular events [17, 18]. Integrins are heterodimers composed of two transmembrane proteins, namely, *α* and *β* subunits [19]. The *α* and *β* extracellular domains cooperatively bind to extracellular matrix components such as collagen, laminin, and fibronectin. Interactions between integrins and the extracellular matrix modulate essential aspects of cell behavior crucial to the development and maintenance of organisms. In this review, I summarize what is known regarding Hox downstream targets, focusing on molecules mediating cell-cell and cell-extracellular matrix interactions in* Drosophila* and mammals (Table 1). Furthermore, potential roles for Hox proteins in cell adhesion and migration during vertebrate development will be discussed.

## 2. Structural and Functional Organization of* Hox* Genes in* Drosophila* and Mammals

**Figure 1:**
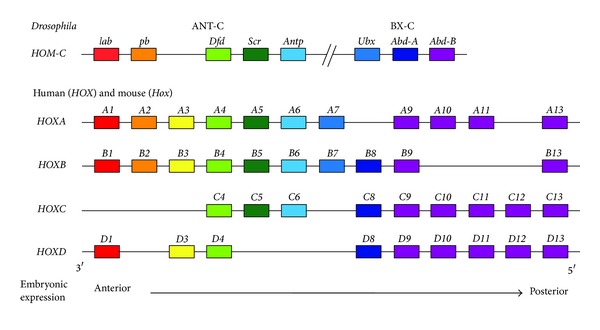
Arrangement of* Hox* genes in the* Drosophila* and mammalian genomes. In* Drosophila*, eight* Hox* genes clustered on a single chromosome, the homeotic complex (*HOM-C*), are divided into two groups: the Antennapedia complex (ANT-C) and Bithorax complex (BX-C). ANT-C comprises five* Hox* genes:* labial* (*lab*),* proboscipedia* (*pb*),* Deformed* (*Dfd*),* Sex combs reduced* (*Scr*), and* Antennapedia *(*Antp)*. The BX-C consists of three* Hox* genes:* Ultrabithorax* (*Ubx*),* Abdominal-A* (*Abd-A*), and* Abdominal-B* (*Abd-B*). In mammals, 39* Hox* genes are divided into four separate clusters (*HoxA*,* HoxB*,* HoxC*, and* HoxD*) on four different chromosomes. In each cluster,* Hox* genes are tandem arranged in sequence from 3′ to 5′.* Hox* genes with the same number are referred to as paralogs. In the embryo, expression of the 3′ paralogs occurs earlier and more anteriorly along the anterior-posterior axis, whereas the 5′ paralogs are expressed later and more posteriorly.

In* Drosophila*, eight* Hox* genes are clustered in two groups: the Antennapedia complex (ANT-C) and Bithorax complex (BX-C) (Figure 1). The order of genes along the chromosome corresponds to their domains of function along the anterior-posterior axis of the animal. The* labial* (*lab*) and* Deformed* (*Dfd*) genes specify the head segments, while* Sex combs reduced* (*Scr*) and* Antennapedia* (*Antp*) are required for the identities of the first and second thoracic segments, respectively.* Ultrabithorax* (*Ubx*) is responsible for specifying third thoracic segment identity, and* Abdominal A* (*Abd-A*) and* Abdominal B* (*Abd-B*) contribute to specifying abdominal segment identities. In homeotic mutants, these specific segmental identities can be changed. For example, a loss-of-function mutation in* Ubx* gives rise to flies with two sets of wings, due to the transformation of the third thoracic segment into one with second thoracic segment identity. This transformation, referred to as “anteriorization,” is caused by the functional substitution of the more anterior gene* Antp* for* Ubx*.

In mammals, 39* Hox* genes are organized in four different clusters (*HoxA*,* HoxB*,* HoxC*, and* HoxD*) found at four distinct chromosomal loci (Figure 1). These clusters are thought to have arisen by two duplication events during the emergence of the vertebrates. Based on the nucleotide sequence similarities between the* Hox* genes and their* Drosophila* counterparts, these genes are classified into 13 homology groups, referred to as paralogs [20]. As observed in* Drosophila*, the order of these paralogs on their respective chromosomes shows collinearity with the spatiotemporal expression pattern of these genes in the embryo [21].* Hox* expression can be seen in the neural tube, neural crest, paraxial mesoderm, and surface ectoderm, along the anterior-posterior axis. The 3′* Hox* genes are expressed more anteriorly and earlier, while the 5′* Hox* genes are expressed more posteriorly and later [22, 23]. Morphological analyses of* Hox* knockout mice show that the segmental identity of the body along the anterior-posterior axis is primarily determined by the posterior-most* Hox* gene expressed in the segment [24]. Disruption of all* Hox10* paralogs results in the conversion of lumbar vertebrae into thoracic vertebra-like structures with rib projections. Similarly, when all* Hox11* paralogs are deleted, sacral vertebrae are transformed into vertebrae with lumbar identity [25]. Thus, homeotic transformations comparable to those in* Drosophila* occur in mutant mice that are null for all the paralogs belonging to a particular group. To directly investigate how* Hox* cluster duplications contributed to morphological innovations in vertebrates during evolution, mutant mouse embryos, in which full* Hox* clusters are deleted, have been generated. Mice lacking all HoxA and HoxD functions in their forelimbs show an early developmental arrest of the limbs and severe truncations of distal elements, suggesting that the evolutionary recruitment of Hox proteins into growing appendages leads to distal extension of tetrapod appendages [26]. Deletion of both* HoxA* and* HoxB* clusters results in a heart-looping defect that is recognized as an atavistic phenotype, suggesting that both* HoxA* and* HoxB* clusters were necessary for vertebrate heart evolution [27]. In addition, a growing body of recent work highlights the significance of functional organization of* Hox* gene clusters in vertebrate evolution [28–32].

## 3. Cell Adhesion Molecules Identified as Hox Realizators during Segment-Specific Organogenesis in* Drosophila*

In* Drosophila*, 17 different proteins that contain cadherin domains have been identified. Of these, E-cadherin and two N-cadherins are considered classical types, while the remaining 14 cadherins are regarded as nonclassical cadherins [35]. In addition,* Drosophila* has 5 integrin *α* subunits (*α*PS1–5) and 2 integrin *β* subunits (*β*PS and *β* *ν*) [36]. These cell adhesion molecules play versatile roles in the development and adult life of* Drosophila* and interact with cytoplasmic proteins to form adhesion complexes that link their intracellular domains with the cytoskeleton [37].

Posterior spiracles connect the tracheal respiratory systems of* Drosophila* larvae to the external environment. The* Hox* gene* Abd-B* is required to induce the specification and morphogenetic movements required for posterior spiracle formation, as evidenced by the lack of spiracles in* Abd-B* mutants and formation of ectopic spiracles when* Abd-B* is ectopically expressed [38, 39]. A study by Lovegrove et al. [40] has provided a framework for understanding how* Abd-B* controls posterior spiracle formation. Abd-B activates three transcription factors,* spalt* (*sal*),* empty spiracle* (*ems*), and* cut* (*ct*), and a signaling molecule, unpaired (*upd*, the ligand of the JAK/STAT pathway), the expression of which leads to the activation of realizator molecules controlling cell adhesion. The Abd-B direct target Ct promotes the E-cadherin expression that is responsible for ectodermal cell invagination during the formation of the spiracular chamber, the internal tube connecting the trachea to the exterior of the larva. The expression of four nonclassical cadherins in different spiracle cell domains is controlled by several regulators (Sal, Ems, Ct, and Upd) that partially overlap in expression. E-cadherin and nonclassical cadherins cooperate to control spiracle cell invagination, suggesting that these adhesive molecules, which function in the Abd-B-regulated molecular cascade, play crucial roles in spiracle organogenesis.

The salivary gland is a simple tubular organ composed of two major cell types: secretory and duct cells [58]. The Hox protein Scr, which forms a transcriptional complex with the extradenticle and homothorax homeodomain proteins, is required for salivary gland formation, as evidenced by the complete absence of salivary glands because of* Scr* loss of function [41]. Although Scr is critical for the specification of salivary gland fates, the protein cannot directly maintain salivary gland cell identity because it disappears early in salivary gland development [58]. Once specified, the salivary gland primordium forms a placode of columnar epithelial cells within the ventral ectoderm [42]. The *α* *PS1* gene, which encodes an integrin *α* subunit, is expressed in the salivary gland primordium formed within the ventral ectoderm. At later embryonic stages, *α* *PS1* expression is maintained in invaginating and posteriorly migrating secretory cells that keep in contact with the visceral mesoderm substratum. Embryos carrying* Scr* mutations lack *α* *PS1* expression in the salivary primordium, suggesting that *α* *PS1* is a downstream target of Scr [42]. In *α* *PS1* mutants, the distal tip of invaginating secretory cells reaches the turning point of the visceral mesoderm, but these cells fail to migrate posteriorly [41]. These salivary gland defects, observed when Scr and *α*PS1 expression is lost, suggest that integrin *α*PS1 participating in the Scr-directed molecular cascade is essential for the salivary gland to migrate posteriorly along the visceral mesoderm.

## 4. Hox-Regulated Cell Adhesion Molecule Expression in Cultured Normal and Cancer Cells

The neural cell adhesion molecule (N-CAM), a member of the immunoglobulin superfamily, is involved in cell adhesion, intracellular signaling, and cytoskeleton dynamics [59]. The effects of Hox proteins on* N-CAM* promoter activity have been investigated by cotransfecting NIH 3T3 mouse embryonic fibroblasts with constitutively active* Xenopus Hox* constructs and a reporter gene construct containing the mouse* N-CAM* promoter sequence.* Hox2.5* (*Hoxb9*) greatly increases the transcriptional activity of the reporter gene, while transfection of* Hox2.4* (*Hoxb8*) eliminates its activity [16].* Hoxc6* also stimulates the transcriptional activity driven by the* N-CAM* promoter [49]. Together, these findings suggest that* N-CAM* is a downstream target for regulation by Hoxb8, Hoxb9, and Hoxc6.

*HOXD3* overexpression in human erythroleukemia HEL cells results in an increase of cell-extracellular matrix adhesiveness, giving rise to elevated *β*3 integrin expression levels [45, 46]. Human lung carcinoma A549 epithelial cells transfected with* HOXD3* exhibit an increase in *β*3 integrin expression and this modification promotes migratory and invasive behavior [33, 60].* HOXD3* expression elicits phenotypic changes in human umbilical vein endothelial cells (HUVECs), switching them from a resting to angiogenic or invasive state by enhancing *α*v*β*3 integrin expression [47]. HOXD3 directly binds to the *β* *3 integrin* promoter in human microvascular endothelial cells [61]. While HOXD3 causes an increase in *β* *3 integrin* expression in several cell lines, HOXB3, which is paralogous to HOXD3, is not involved in *β* *3 integrin* expression in endothelial cells [62]. Although the HOXA3 paralog is functionally similar to HOXD3 with respect to promotion of cell migration, these transcription factors do not have common downstream target genes [63].  *β* *3 integrin* mRNA levels are increased in endometrial adenocarcinoma cells transfected with a* HOXA10* expression vector and are decreased in the cells treated with a * HOXA10* antisense construct [54]. HOXA10 directly regulates *β* *3 integrin* expression in endometrial cells, mediating the effects of steroid hormones, estrogen and progesterone, on *β* *3 integrin* expression [54]. The HOXA10 transcription factor interacts with a specific *β* *3 integrin* cis element, activating *β* *3 integrin* transcription during differentiation of U937 cells into a myeloid lineage [55]. Increased adhesion of differentiating U937 cells to fibronectin is dependent upon a HOXA10-induced increase in *β*3 integrin expression [55]. Thus, expression of *β* *3 integrin* can be controlled by at least two HOX proteins that belong to different paralogous groups, possibly reflecting the redundant functions of the different HOX paralogs.

In addition to *β*3 integrin, expression of several integrins is reportedly regulated by HOX transcription factors. An approximate 20-fold increase in *α* *8 integrin* expression levels is caused by ectopic* Hoxa11* expression in human embryonic kidney 293 cells [56]. During development,  *α* *8 integrin *and* Hoxa11* are coexpressed in mouse metanephric mesenchyme cells. Mutations in the *α* *8 integrin *gene give rise to a bud branching morphogenesis defect that is very similar to that observed in* Hoxa11*/*Hoxd11* mutant mice. Furthermore, a regional reduction in *α* *8 integrin *expression is found in the developing kidneys of * Hoxa11*/*Hoxd11* mutant mice [56]. These findings suggest that *α* *8 integrin *is a major realizator of * Hoxa11*/*Hoxd11* function in the developing kidney.

In ovarian cancer epithelial cells, HOXA4 suppresses cell motility and spreading through the medium by increasing cell-cell adhesion and *β*1 integrin protein levels [48]. Loss of HOXD1 expression in HUVECs results in a decrease in cell motility and cell-extracellular matrix adhesiveness, accompanied by decreasing *β*1 integrin expression levels, suggesting HOXD1 is a positive regulator of cell motility and cell-extracellular matrix adhesiveness in endothelial cells [44]. Thus, it is possible that cell-extracellular matrix interactions mediated by different types of integrin molecules are dependent on the assortment of* HOX* genes expressed and the amount of protein they produce in nonmalignant and malignant cells.

## 5. A Role for Hox Proteins in Epithelial to Mesenchymal Transition and Its Reverse Process in Normal and Cancer Cells

Epithelial to mesenchymal transition (EMT) is an event in which adherent epithelial cells are converted into migratory mesenchymal cells that can invade the extracellular matrix. The EMT process is essential for gastrulation and neural crest migration during the development of the early vertebrate embryo. EMT also plays a role in cancer metastasis. Mesenchymal to epithelial transition, the converse of EMT, is observed in many aspects of embryonic development and tumor metastasis, suggesting that epithelial and mesenchymal morphologies are reversible [64].

*HOX* expression is reported to be closely associated with the transition between epithelial and mesenchymal states.* HOXA7* transcripts are absent from normal ovarian surface epithelial cells, but HOXA7 protein is produced in ovarian tumors derived from epithelial cells, which often resemble epithelia composing the Müllerian duct. Ectopic* HOXA7* expression in immortalized ovarian surface epithelial (IOSE-29) cells induces E-cadherin expression and downregulates expression of the mesenchymal marker vimentin, enhancing the epithelial phenotype [50].* Hoxa10* is required for proper patterning of the uterus during embryonic development and functional endometrial differentiation in adults [65]. Downregulation of* HOXA10* expression in endometrial carcinomas correlates with increased tumor grade and promotes tumor growth and invasive properties [53]. Forced expression of* HOXA10* in endometrial carcinoma (SPEC2 and KLE) cells induces E-cadherin expression, suppresses vimentin expression, and inhibits their invasive behavior [53]. The findings described above suggest that* HOXA7* and* HOXA10* expression promotes mesenchymal to epithelial transition.

**Figure 2:**
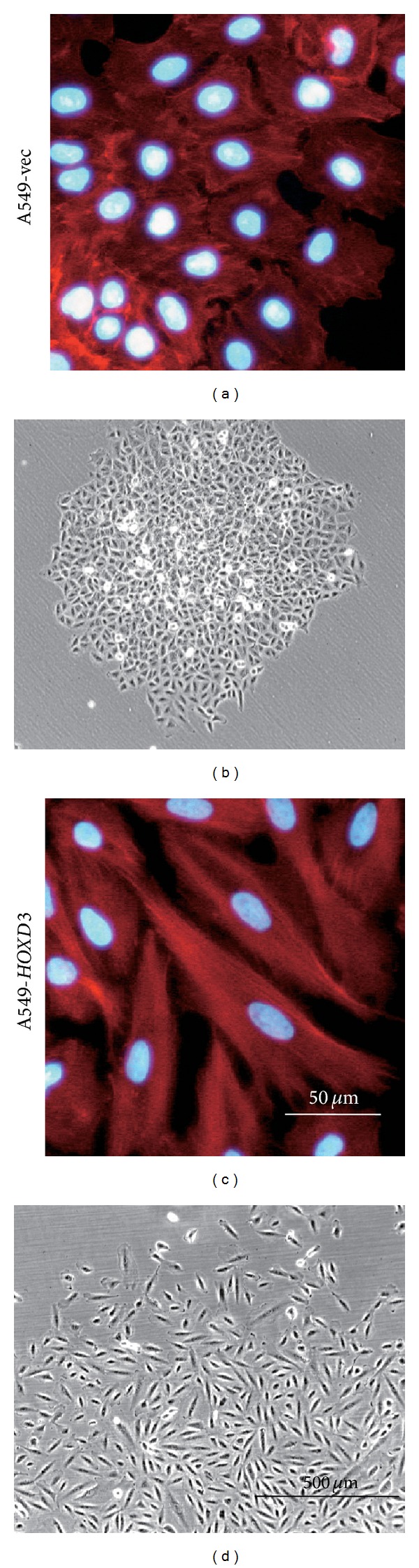
Transition from epithelial to mesenchymal morphology caused by* HOXD3* expression in lung cancer A549 cells. A549 cells stably transfected with empty vector (A549-vec) or* HOXD3* expression vector (A549-*HOXD3*) [33] were fixed and stained for nuclei and F-actin by using DAPI and phalloidin-rhodamine, respectively. A549-vec cells have epithelial morphology (a, b), while A549-*HOXD3* cells have spindle-shape mesenchymal morphology (c, d). A reduction in E-cadherin expression and an increase in *α*3 and *β*3 integrin expression were observed in A549-*HOXD3* cells, as compared to A549-vec cells [33].

In contrast,* HOXD3* overexpression in lung cancer A549 cells transforms them from epithelial to mesenchymal morphology (Figure 2) and causes a simultaneous reduction in E-cadherin expression levels and increase in *α*3 and *β*3 expression [33]. This was the first study reporting that* HOX* gene expression enhances the invasive and metastatic properties of human cancer cells. Primary breast carcinomas and distant metastases of various organs exhibit significantly higher* HOXB7* expression levels than normal mammary epithelial cells [51]. Overexpression of* HOXB7* in MCF10A cells, an immortalized cell line derived from normal human mammary epithelial cells, induces their transformation from cobblestone-like epithelial morphology to spindle-shape mesenchymal morphology, which brings about a dramatic reduction in expression of E-cadherin and tight junction proteins, claudin 1, claudin 4, and claudin 7, as well as an elevation in *α*-smooth muscle actin expression [51]. Similarly,* HOXB9* overexpression in MCF10A cells transforms them from an epithelial phenotype into a mesenchymal phenotype by reducing E-cadherin expression levels and increasing vimentin expression [52]. These findings suggest that HOXD3, HOXB7, and HOXB9 transcription factors serve as EMT inducers in immortalized cells and cancer cells.

Whether EMT-inducing HOX proteins have the ability to regulate adhesion molecule gene expression directly or where in the signal transduction pathway HOX proteins exert their effect to induce EMT warrants clarification. HOX proteins have been reported to control expression of some regulatory molecules. HOXA10 inhibits expression of Snail, a zinc-finger transcription factor, in endometrial carcinoma cells [53]. Snail, a key regulator of EMT, downregulates E-cadherin expression, leading to the loss of epithelial morphology in cells undergoing migration during embryonic development as well as tumor progression [66–68]. These results clearly suggest that downregulation of* HOXA10* expression induces EMT by elevating Snail expression levels. HOXB9 induces elevated expression of signaling molecules, TGF-*β*1 and TGF-*β*2, in MCF10A cells, leading to increased cell motility and acquisition of mesenchymal phenotypes [52]. Members of the TGF-*β* family play crucial roles in initiating and maintaining EMT during embryonic development and tumor metastasis [69, 70]. These findings indicate that* HOXB9* expression induces EMT by activating the TGF-*β* signaling pathway.

During development of the vertebrate embryo, neural crest cells initially reside within the dorsal neural tube, subsequently undergo EMT to migrate to distant locations, and then differentiate into a wide range of derivatives. When neural crest cells delaminate from the neuroepithelium, N-cadherin and cadherin 6B are downregulated and *β*1 integrin and cadherin 7 are upregulated [71]. The EMT process is controlled by a hierarchical gene regulatory network in which transcription factors and signaling molecules operate [72]. A recent study [43] has demonstrated that anterior* Hox* genes interact with components of this network to induce neural crest fates in the chick embryo. Expression of* Hoxb1* in the trunk neural tube induces expression of the key transcription factors Snail and Msx1/2, leading to downregulation of N-cadherin and cadherin 6B expression and upregulation of cadherin 7. These changes in cell adhesion molecule expression possibly reflect that Hoxb1 causes neural crest EMT. It is interesting to note that expression of* Hox* genes participates in EMT events that occur during embryonic morphogenesis as well as tumor progression.

## 6. Possible Association between* Hox* Expression and Cell-Cell and Cell-Extracellular Matrix Interactions in the Vertebrate Embryo during Development

**Figure 3:**
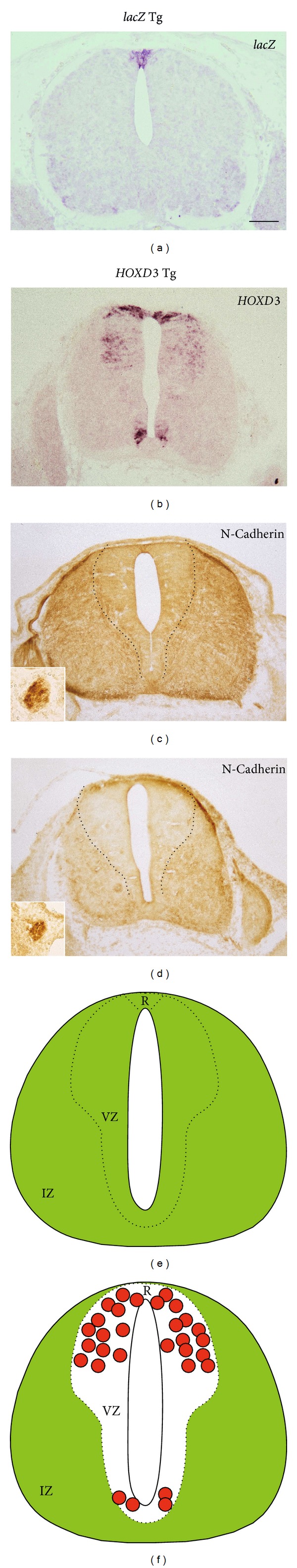
Reduced N-cadherin expression induced by* HOXD3* overexpression in the roof plate of the early mouse embryo. (a, b) Expression of* lacZ* and* HOXD3* genes in transverse neural tube sections at the thoracic level of 12.5-day transgenic embryos. Transgenic embryos were generated, in which* lacZ* and* HOXD3* are expressed in the roof plate cells under the control of the* Wnt1* regulatory element [34]. These embryos were sectioned and analyzed using in situ hybridization. Expression of* lacZ* (control) is restricted to roof plate cells within the neural tube, while* HOXD3* expression is localized not only in the dorsal neural tube, but also within the ventricular zone and in ventral regions of the neural tube. (c, d) N-Cadherin expression in the thoracic neural tubes of 12.5-day* lacZ*- and* HOXD3*-expressing transgenic embryos. Transverse sections were stained using anti-human N-cadherin antibodies [34]. N-Cadherin is strongly expressed in the ventricular zone of* lacZ*-expressing embryos, whereas the ventricular zone in* HOXD3*-expressing embryos is composed of a number of progenitor cells that do not express N-cadherin. The ventricular zone is surrounded by dotted lines. Insets show that N-cadherin expression levels in the sympathetic ganglia of* lacZ*-expressing embryos are similar to those of* HOXD3*-expressing embryos. (e, f) Summary of the neural tube phenotype in transgenic embryos expressing* lacZ* and* HOXD3*. In embryos expressing* lacZ*, N-cadherin expression (green) is distributed throughout the neural tube. In* HOXD3*-expressing embryos, roof plate cells expressing* HOXD3* (red circles) expand ventrally into the ventricular zone, where almost all N-cadherin-expressing cells are lost. R, roof plate; IZ, intermediate zone; VZ, ventricular zone. Scale bar: 100 *μ*m.

When neural crest cells delaminate from the dorsal neural tube by EMT, these cells lose N-cadherin on their surfaces. [73–75]. As mentioned previously, HOXD3 promotes cell motile activity and invasiveness in lung cancer cells [33]. To investigate whether* HOXD3* expression regulates cell adhesiveness in dorsal neural tube or roof plate cells in the early mouse embryo, transgenic mouse embryos were generated that overexpress* HOXD3* in these cell types under the control of the* Wnt1* regulatory element [34]. Dorsal neural tube cells expressing* HOXD3* expand ventrally within the neural tube (Figures 3(a), 3(b), 3(e), and 3(f)). This finding raises the possibility that* HOXD3*-expressing roof plate cells propagate in the dorsal neural tube and then migrate ventrally. Furthermore, in the neural tube ventricular zone, a large number of progenitor cells that do not express N-cadherin protein can be observed in* HOXD3*-expressing transgenic embryos (Figures 3(c), 3(d), 3(e), and 3(f)). Although* HOXD3* expression is localized in the dorsal half of the neural tube and in cells immediately adjacent to the floor plate, progenitor cells that do not express N-cadherin are distributed throughout the ventricular zone. This finding indicates that* HOXD3* expression has a non-cell-autonomous effect, negatively affecting N-cadherin expression in cells at a distance from those expressing* HOXD3*. Therefore, signaling molecules or secreted proteins whose expression is induced by HOXD3 likely reduce N-cadherin expression.

Gastrulation is an essential process in the development of most animals. In amniotes, gastrulation begins with the acquisition of asymmetry in the early embryo. The movements of epiblast cells towards the midline of the embryo form the primitive streak. At the streak, epiblast cells undergo EMT, ingress, and migrate inwardly to their proper positions where they differentiate into mesodermal and endodermal tissues. Consequently, the three definitive germ layers, ectoderm, mesoderm, and endoderm, are organized. The crucial role of FGF signaling in regulating cell migration is highlighted by the effect of altering* fibroblast growth factor receptor 1* (*FGFR1*) expression. In* Fgfr1*-deficient mouse embryos, epiblast cells fail to undergo EMT, which is required for ingression through the primitive streak [76]. The defect is attributed to a failure in* Snail* upregulation and E-cadherin downregulation. This finding shows that FGFR1 regulates epiblast cell migration by differentially regulating the intercellular adhesion properties of these cells at the primitive streak. Furthermore, this study suggests that* Snail* expression downstream of FGFR1 is required for normal downregulation of E-cadherin. In the early chick embryo, PDGF signaling plays a major role in the migration of mesodermal cells during gastrulation [77]. PDGFA expression in the epiblast controls N-cadherin expression and activates PDGFR*α*, which is required for migration of mesodermal cells away from the primitive streak. The timing of ingression is orchestrated by temporal and spatial collinear activation of* Hox* genes that starts in the epiblast [78]. Expression of posterior* Hox* genes can delay the time at which cells ingress from the epiblast into the primitive streak and nascent mesoderm. Within a region of epiblast cells expressing a given* Hox* gene, a subpopulation of epiblast cells that express the neighboring 5′* Hox* gene exists. These cells acquire slightly different migratory properties, and their ingression is slightly delayed. Ingressing cells expressing* Hox* genes from successive paralogous groups might sort out from each other along the anterior-posterior axis [24, 78]. The target genes of Hox proteins and the mechanism by which they control ingression remain to be elucidated; however, the targets might include genes encoding factors that regulate EMT, such as cell-cell and cell-extracellular matrix adhesion molecules [79].

In the developing mouse embryo,* Hox3* paralogs play crucial roles in the formation of neural crest, somatic mesoderm, and endoderm-derived structures in the cervical region, including the pharyngeal arches [80, 81].* Hoxa3* is essential for the development of the thymus, thyroid, parathyroid glands, and ultimobranchial bodies [82]. These organs develop concurrently and they are composed of cells that migrate from their original sites in the pharynx and pharyngeal pouches to their final positions in the cervical and upper thoracic regions. The ultimobranchial bodies fuse with the thyroid; the cells disperse within the thyroid lobes and then differentiate into calcitonin-producing C-cells. Mice doubly mutant for* Hoxa3* and* Hoxb3* or* Hoxa3* and* Hoxd3* show that the ultimobranchial bodies fail to migrate to their normal positions in the thyroid, suggesting that expression of* Hox3* paralogs is required for the organized movement of primordial organs in the pharyngeal tissues [83]. The thymus and parathyroid glands originate from both the neural crest-derived mesenchymal cells of the pharyngeal arches and the pharyngeal endoderm. Conditional deletion of* Hoxa3* alleles from neural crest cells results in the development of ectopic thymus and parathyroid glands [84], raising the possibility that Hoxa3 controls neural crest cell migration in pharyngeal regions. In the chick embryo, knockdown of* Hoxa3* function by using antisense morpholino oligonucleotides disrupts the migration of epibranchial placode-derived cells and neural crest cells, indicating that Hoxa3 is required for the migration of these cell types [85]. Although these findings show that Hoxa3 and its paralogs are regulators of cell migration, the target genes for Hox3 proteins are not known. Genes encoding molecules involved in regulating cell-cell and cell-extracellular matrix interactions could be candidate Hox3 paralog targets.

During vertebrate limb development, posterior* Hox* genes in the* HoxA* cluster are expressed in a specific spatiotemporal manner along the proximodistal axis.* Hoxa13* is expressed in the autopod during normal limb development. In the chick embryo, misexpression of* Hoxa13* in the entire limb bud results in a marked size reduction of the zeugopodal cartilage due to homeotic transformation into cartilage of a more distal type [86]. When limb mesenchymal cells are dissociated and cultured in vitro,* Hoxa13*-expressing cells sort out from* Hoxa13*-nonexpressing cells. This finding indicates that* Hoxa13* expression is involved in modulation of cell-cell adhesiveness. Mice homozygous for a* Hoxa13 *loss-of-function mutation show major defects in the formation of autopod skeletal elements [87]. Autopod-derived mesenchymal cells in homozygous* Hoxa13* mutant embryos fail to form chondrogenic condensations in vitro, and mutant cells in the distal region fail to sort out from wild-type cells in the proximal region [57]. This failure in cell sorting reflects the fact that* Hoxa13* expression is involved in determining cell surface properties. Eph proteins, which constitute a large family of receptor tyrosine kinases, interact with cell surface-bound ligands, ephrins [88, 89]. Eph/ephrin juxtacrine signaling modulates cell morphology, motility, and attachment. A marked reduction in* EphrinA7* expression prevents mesenchymal cells in the autopod of homozygous* Hoxa13* mutant embryos from forming chondrogenic condensations in vivo and in vitro [57].* EphrinA7* has been shown to be a direct downstream target of Hoxa13 and Hoxd13 during limb development [90]. Furthermore, using a CHIP-on-chip approach (chromatin immunoprecipitation with DNA microarray technology), the gene loci of* cadherin 12* (also known as* Br-cadherin* or* N-cadherin 2*) and* protocadherins* are identified as direct Hoxd13 binding sites in the developing mouse limb bud [91]. It has been reported that the cadherin 12 protein is exclusively expressed in the developing and adult mouse brain [92, 93]. Cadherin 12 does not seem to function in the limb bud. On the other hand, N-cadherin is abundant in the distal limb bud and increases in the distal region as limb development proceeds [94, 95]. N-Cadherin-positive mesenchymal cells segregate from N-cadherin-negative cells in vitro, suggesting that N-cadherin plays an important role in cell sorting. However, the relation between N-cadherin and expression of* Hox* genes during limb development is presently unknown.

## 7. Concluding Remarks

In this review, cell adhesion molecules mediating cell-cell and cell-extracellular matrix interactions, whose expression is directly or indirectly controlled by Hox transcription factors, have been the focus. In* Drosophila*, cadherins, components of the hierarchical Abd-B-regulated molecular pathway, play an important role in the formation of posterior spiracles during development. Integrin molecules participate in the Scr-directed molecular cascade that is required for salivary gland formation and migration. In cultured normal and malignant mammalian cells, expression of several* Hox* genes enhances cell-extracellular matrix adhesion and cell motility by activating* integrin *expression. Several Hox proteins play a role in epithelial-mesenchymal transition and its reverse process by reducing and elevating cadherin expression. Hox proteins likely do not regulate cadherin expression directly; Hox proteins might control cadherin expression by using transcription factors and signaling molecules as intermediaries. To elucidate the exact processes governed by Hox proteins, it is worthwhile to investigate whether cell adhesion molecule expression is directly controlled by Hox proteins or where in the Hox-directed molecular cascade cell adhesion molecules function.

In this review, I have discussed the necessity of* Hox* expression for neural crest migration, gastrulation, migration of organs in the pharyngeal regions, and limb bud formation in the vertebrate embryo during development. These developmental processesrequire precise regulation of cell adhesion and migration. How Hox proteins are related to expression of cell adhesion molecules during vertebrate body patterning is not fully understood. The highly redundant functions of* Hox* genes pose a challenge when attempting to clarify the association between Hox transcription factors and expression of a diverse set of cell adhesion molecules. However, as the gaps in the puzzle are filled by future research findings, the precise mechanisms by which Hox proteins govern expression of cell adhesion molecules will be uncovered.

## Figures and Tables

**Table 1:** Hox proteins controlling expression of cell adhesion molecules.

Hox protein	Controlled cell adhesion molecules	Organism	Cell or tissue	Proposed function	References
Scr	Integrin *α* subunit (*α*PS1) (+)	*Drosophila *	Salivary gland	Organ formation and migration	[41, 42]

Abd-B	E-Cadherin (+) Nonclassical cadherins (+)	*Drosophila *	Posterior spiracle	Organ formation	[40]

Hoxb1	N-Cadherin (−) Cadherin 6B (−) Cadherin 7 (+)	Chick	Neural crest cells	EMT	[43]

HOXD1	*β*1 Integrin (+)	Human	HUVEC	Increase in cell motility	[44]

Hoxa2	Cadherin 6B (−)	Chick	Neural crest cells	EMT	[43]

HOXD3	*β*3 Integrin (+) Cadherin 4 (−)	Human	Erythroleukemia (HEL)	Increased adhesion to fibronectin	[45, 46]

HOXD3	*β*3 Integrin (+)	Human	HUVEC	Conversion to angiogenic phenotype	[47]

HOXD3	*α*3 Integrin (+) *β*3 Integrin (+) E-Cadherin (−) N-Cadherin (+)	Human	Lung cancer (A549)	Increase in cell motility EMT	[33]

HOXD3	N-Cadherin (−)	Mouse	Roof plate cells	Expansion in the neural tube	[34]

HOXA4	*β*1 Integrin (+)	Human	Ovarian cancer epithelium	Decrease in cell motility	[48]

Hoxc6	N-CAM (+)	Mouse	NIH 3T3 fibroblast	Increase in promoter activity	[49]

HOXA7	E-Cadherin (+)	Human	Ovarian surface epithelium (IOSE-29)	MET	[50]

HOXB7	E-Cadherin (−) claudin 1, 4, 7 (−)	Human	Mammary epithelium (MCF10A)	EMT	[51]

Hoxb8	N-CAM (−)	Mouse	NIH 3T3 fibroblast	Reduction in promoter activity	[16]

Hoxb9	N-CAM (+)	Mouse	NIH 3T3 fibroblast	Increase in promoter activity	[16]

HOXB9	E-Cadherin (−)	Human	Mammary epithelium (MCF10A)	EMT	[52]

HOXA10	E-Cadherin (+)	Human	Endometrial carcinoma (SPEC2, KLE)	MET	[53]

HOXA10	*β*3 Integrin (+)	Human	Endometrium	Pathway regulated by sex steroid	[54]

HOXA10	*β*3 Integrin (+)	Human	Myeloma (U937)	Increased adhesion to fibronectin	[55]

HOXA11	*α*8 Integrin (+)	Human	Embryonic kidney 239	Branching morphogenesis	[56]

Hoxa13	EphrinA7 (+)	Mouse	Mesenchyme in limb bud	Cell sorting	[57]

(+): upregulated expression; (−): downregulated expression; HUVEC: human umbilical vein endothelial cells; MET: mesenchymal to epithelial transition; EMT: epithelial to mesenchymal transition.
